# Land Snail Shell Beads in the Sub-Saharan Archaeological Record: When, Where, and Why?

**DOI:** 10.1007/s10437-018-9305-3

**Published:** 2018-07-30

**Authors:** Jennifer M. Miller, Elizabeth A. Sawchuk, Amy L. R. Reedman, Pamela R. Willoughby

**Affiliations:** 1 Department of Anthropology, University of Alberta, Edmonton, Alberta Canada; 2 Department of Anthropology, Stony Brook University, New York, NY USA

**Keywords:** *Achatina*, Land snail, Shell bead, Iron Age, Later Stone Age

## Abstract

Shell beads are well established in the archaeological record of sub-Saharan Africa and appear as early as 75,000 BP; however, most research has focused on ostrich eggshell (OES) and various marine mollusc species. Beads made from various land snails shells (LSS), frequently described as *Achatina*, also appear to be widespread. Yet tracking their appearance and distribution is difficult because LSS beads are often intentionally or unintentionally lumped with OES beads, there are no directly dated examples, and bead reporting in general is highly variable in the archaeological literature. Nevertheless, *Achatina* and other potential cases of LSS beads are present at over 80 archaeological sites in at least eight countries, spanning the early Holocene to recent past. Here, we collate published cases and report on several more. We also present a new case from Magubike Rockshelter in southern Tanzania with the first directly dated LSS beads, which we use to illustrate methods for identifying LSS as a raw material. Despite the long history of OES bead production on the continent and the abundance of land snails available throughout the Pleistocene, LSS beads appear only in the late Holocene and are almost exclusively found in Iron Age contexts. We consider possible explanations for the late adoption of land snails as a raw material for beadmaking within the larger context of environmental, economic, and social processes in Holocene Africa. By highlighting the existence of these artifacts, we hope to facilitate more in-depth research on the timing, production, and distribution of LSS beads in African prehistory.

## Résumé

Les perles de coquillages sont bien établies dans les archives archéologiques de l’Afrique subsaharienne et apparaissent dès 75 000 BP, mais la plupart des recherches ont porté sur la coquille d’œuf d’autruche (OES) et diverses espèces de mollusques marins. Les perles fabriquées à partir de diverses coquilles d’escargots terrestres (LSS), souvent décrites comme *Achatina*, semblent également être répandues. Cependant, le suivi de leur apparence et de leur distribution est. difficile parce que les billes LSS sont souvent volontairement ou involontairement regroupées avec des billes OES, il n’y a pas d’exemples directement datés, et les rapports sur les perles en général sont très variables dans la littérature archéologique. Néanmoins, *Achatina* et d’autres cas potentiels de perles LSS sont présents sur plus de 80 sites archéologiques dans au moins huit pays, couvrant le début de l’Holocène à un passé récent. Ici, nous rassemblons les cas publiés et rapportons plusieurs autres cas. Nous présentons également un nouveau cas de Magubike Rockshelter dans le sud de la Tanzanie avec les premières perles LSS directement datées, que nous utilisons pour illustrer les méthodes d’identification du LSS comme matière première. Malgré la longue histoire de la production de perles d’OES sur le continent et l’abondance d’escargots terrestres disponibles tout au long du Pléistocène, les perles de LSS n’apparaissent qu’à la fin de l’Holocène et sont presque exclusivement trouvées dans des contextes de l’âge du fer. Nous considérons les explications possibles de l’adoption tardive des escargots terrestres en tant que matière première pour la fabrication de perles dans le contexte plus large des processus environnementaux, économiques et sociaux en Afrique de l’Holocène. En mettant en évidence l’existence de ces artefacts, nous espérons faciliter des recherches plus approfondies sur le calendrier, la production et la distribution des perles LSS dans la préhistoire africaine.

## Introduction

Shell beads have a long history of production in sub-Saharan Africa and are one of the first indicators of early modern human symbolic behavior, appearing by 75,000 years ago alongside other forms material culture such as utilized ochre and portable/parietal art (d’Errico et al. 2005; Henshilwood and Marean 2003; McBrearty and Brooks 2000; Wadley 2001). Although the earliest examples were perforated whole marine shells, standardized production of shaped ostrich eggshell (OES) beads was established by at least 50,000 BP (Miller and Willoughby 2014). OES beads remain well represented in Later Stone Age (LSA) and Iron Age (IA) deposits, with the tradition continuing into the ethnographic present among linguistically and culturally diverse communities (Chittick 1975; Lee 1984; Marshall 1976; Silberbauer 1965, 1981; van der Sleen 1958). In contrast to more extensive research on glass beads, however, few studies have moved beyond quantification of OES to focus on chronology, distribution, and manufacture. Such studies are typically focused on metric analyses (e.g., Jacobson 1987; Kandel and Conard 2005; Orton 2008; Sadr et al. 2003; Smith et al. 1991, 2001; Wilmsen 2015). Other notable work has drawn on ethnographic data to explore the social contexts of these artifacts (Williams 1987; Wingfield 2009). This paper builds on the OES literature by focusing on a concurrent but even less studied phenomenon: the production of similar disc beads from the shells of terrestrial land snails.

Although land snail shell beads are most often described as *Achatina* in published sources, identified taxa include other genera in the Family Achatinidae (Swainson 1840) such as *Archachatina* (Albers 1860), *Burtoa* (Bourguignat 1889), and *Limicolaria* (Schumacher 1817). Referring to all cases as *Achatina* may produce the undesired effect of masking variation in species used for beadmaking when comparing sites within and across regions. We therefore propose the new designation land snail shell (LSS) beads to complement reporting convention for OES beads without implying taxonomic homogeneity of molluscs.

Land snails are some of the most pervasive and dense archaeological residues found at sites across Africa. However, interpretation on a case-by-case basis is typically limited to whether their presence is owed to taphonomic “natural” causes (such as self-introduction through burrowing) vs. human agency (such as subsistence behavior or harvesting). Walz (2017, p. 90) argues that this “(non-) treatment” of land snail debris overlooks other possibilities that may inform broader interpretations of site formation, economic and social behavior, and localized environments. Although land snails have been documented as a food source in both archaeological (e.g., Lubell 2004; Mehlman 1979; Shipton et al. 2016) and ethnographic (e.g., Marlowe 2010; Mead 1961) contexts, ethnographic uses also include subsistence and household tools, landscape markers, decorations and personal adornment, and ritual implements (summary in Walz 2017, p. 94). While respecting the limits of ethnographic analogy, it seems unlikely that these other uses are purely historic. To illustrate this point, Walz (2017, p. 92) documents land snail shell beads, scoop/spoon implements, and shallow bowls from Iron Age contexts at Kwa Mgogo and Gonja Maore in northeastern Tanzania. Land snail artifacts are perhaps overlooked more often than OES because snails are expected taphonomic agents and/or ecofacts at many archaeological sites, attracting less attention and scrutiny.

Beyond general inattentiveness to snail shells as forms of material culture, LSS beads are likely underreported for several reasons. Primarily, shell beads of all types are generally understudied in archaeological accounts. Whereas glass beads are frequently employed as proxies in Iron Age research for long-distance trading networks, individual and group wealth, and social stratification (Bvocho 2005; Robertshaw et al. 2010), shell beads are more enigmatic. Aside from obviously exotic cases of marine species traded inland (Mitchell 1996), OES and LSS beads appear in both hunter-gatherer and agropastoralist contexts and are not indicative of any single social process. Consequently, reporting for shell beads is typically limited to tabulation by level and/or raw material, occasionally with summary statistics on diameter, aperture form, and thickness. Other times, shell beads are only reported as present/absent (e.g., Flexner et al. 2008; Garlake 1976). Second, LSS beads closely resemble their OES counterparts in coloration, size, and morphology. Because both types of beads are often found within a single site, LSS beads are easily mischaracterized or simply grouped with OES. Ward and Maggs (1988, p. 407) further attribute misidentifications to inadequate magnification and encrustations on bead surfaces. Finally, because of their small size, collection bias may reduce the number of LSS and other beads collected from survey assemblages or excavations where sediments are not finely sieved.

What is becoming clear, however, is that LSS beads are hardly scarce in the sub-Saharan African archaeological record. We now seek to initiate discourse on the existence and distribution of these artifacts and their role in prehistory. This first requires a review of sites where LSS beads have been identified thus far. We then review several ecological aspects of African land snails as a vehicle for discussing how these animals enter the archaeological record, and what aspects of their physiology affect their utility as a raw material for beadmaking. Here, we build upon earlier scholarship aimed at helping archaeologists distinguish LSS beads from OES and other raw materials. Although it may be possible to assign genus or species-level identifications in some circumstances, we argue that, at minimum, describing this type of disc bead as LSS will help improve reporting and inter-site comparisons. We illustrate our methods using a new case study of LSS beads from Magubike Rockshelter in southern Tanzania, which represent the first directly dated beads of this type. The context of these finds at Magubike evokes broader questions on the nature of these artifacts, starting with when they appear and where they are found. Our ultimate objective is to consider why LSS beads begin to appear alongside, but do not replace, OES beads in many places across sub-Saharan African during the latter stages of the Holocene. We believe this can represent a starting point for more focused study of LSS beads in African archaeology, as part of a broader recognition of land snails as an important component of material culture.

## LSS Beads in the Sub-Saharan African Archaeological Record

**Fig. 1:**
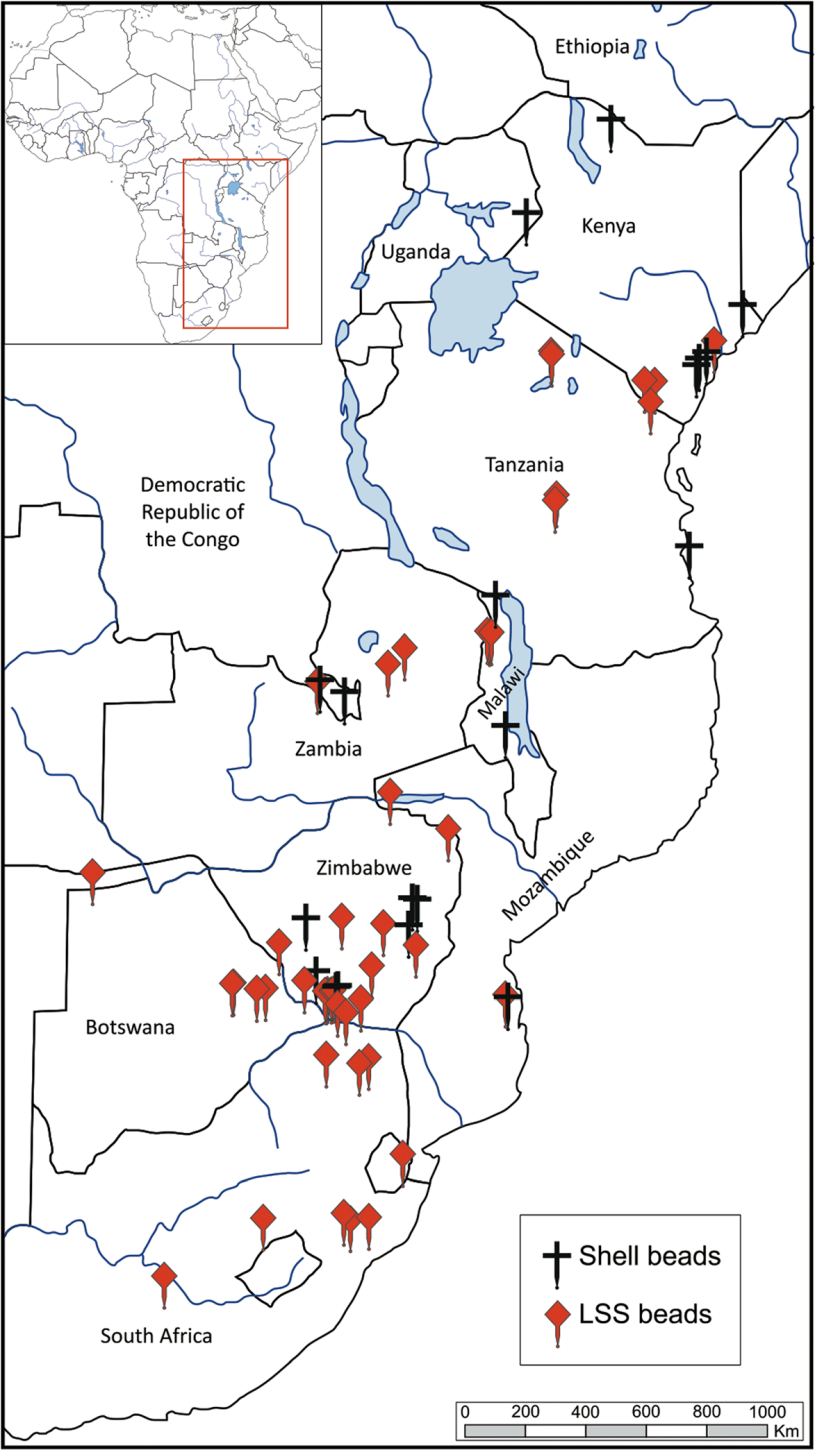
Geographic distribution of sub-Saharan sites with LSS, and potential LSS beads

Reviewing the available literature, it is apparent that LSS beads are not unusual finds. Unfortunately, it also becomes apparent that there is no consistent framework for identification or reporting them. Here, we present the geographic and chronologic distribution of published cases of LSS (and potential LSS) beads (Fig. 1, Table 1). We also include several sites which had not previously published the occurrence of LSS (Mumba, Mlambalasi, Border Cave, and White Paintings Shelter). These cases were ascertained by one of the authors (JM) during first hand observation of collections. It is highly possible that other collections likewise have LSS beads that were mistakenly attributed to OES and have not been further examined.

**Table 1:** List of sub-Saharan archaeological sites with LSS, and potential LSS beads, by country

	Site	Bead Original		Age	Calibrated age (BC/AD)	Reference
		Material	LSS?			
Botswana	Bobonong Road	*Achatina*	y	810 ± 70 BP	659–910 calBP (1,291–1,040 calAD)	Kinahan et al. 1998
	Boutswe	*Achatina*	y	Iron Age	–	DuBroc 2010; Klehm 2013
	Khubu la Dintša	Shell		690 ± 30 BP	563–684 calBP (1,387–1,266 calAD)	Klehm 2013
	Matanga	*Achatina*	y	530 ± 75 BP	338–668 calBP (1,612–1,282 calAD)	Van Waarden 1987
				580 ± 75 BP	508–670 calBP (1,422–1,280 calAD)	
	Mmadipudi Hill	*Achatina*	y	–	990–1,130 calBP (960–820 calAD)	Klehm 2013; Klehm and Ernenwein 2016
					1,390–1,460 calBP (630–490 calAD)	
	Taukome	*Achantina* [sic]	y	995 ± 75 BP	741–1,057 calBP (1,209–893 calAD)	Denbow 1983
				1,240 ± 80 BP	982–1,295 calBP (968–655 calAD)	
				1,265 ± 80 BP	984–1,308 calBP (966–642 calAD)	
	Toutswe (Toutswemogala)	*Achantina* [sic]	y	755 ± 75 BP	554–901 calBP (1,396–1,049 calAD)	Denbow 1983
				990 ± 75 BP	737–1,056 calBP (1,213–894 calAD)	
	White Paintings Shelter	*	y	–	–	Robbins et al. 2000; *
Ghana	Kintampo 6 Rock Shelter	Shell		3,485 ± 100 BP	3,482–4,068 calBP (1,532–2,118 calBC)	Stahl 1985
				3,550 ± 127 BP	3,484–4,226 calBP (1,534–2,276 calBC)	
				3,605 ± 100 BP	3,640–4,225 calBP (1,690–2,275 calBC)	
Kenya	Gede (Gedi)	*Achatina*/shell	y	Iron Age	–	Flexner et al. 2008; Kirkman 1957; Mukhwana 1992
	Kilepwa	Shell		Iron age	–	Kirkman 1952
	Mgombani	Shell		1,300 ± 50 BP	1,304–1,084 calBP (646–866 calAD)	Helm et al. 2012
	North Horr I	Mollusc shell		–	–	Wandibba 1988
	Panga ya Saidi	Shell		Iron Age	–	Helm et al. 2012
	Shanga	Mollusc shell		Iron Age	–	Mukhwana 1992; Robertshaw 1984
	Wabukhe Hill	Mollusc shell		Iron Age	–	Wandibba 1988
Malawi	Fingira	Shell		Later Stone Age	–	Clark 1967
	Hora Mountain	*Achatina*	y	Later Stone Age	–	Clark 1956
	Linthipe/Changoni Sites (DZ40, DZ6A, DZ6B, DZ12, DZ126)	Shell		90 ± 40 BP	12–269 calBP (1,938–1,681 calAD)	Mgomezulu 1978
				1,020 ± 80 BP	739–1,172 calBP (1,211–778 calAD)	
				1,580 ± 80 BP	1,310–1688 calBP (640–262 calAD)	
	Mazinga 1	*Achatina*	y	Iron Age/Later Stone Age	–	Zipkin and Thompson (personal comm.)
	Kadawonda 1	*Achatina*	y	Iron Age/Later Stone Age	–	Zipkin and Thompson (personal comm.)
Mozambique	Chibuene	Shell		Iron Age	–	Sinclair 1982
	Manekweni	*Achatina*	y	340 ± 70 BP	155–514 calBP (1,795–1,436 calAD)	Garlake 1976; Otlet and Walker 1979
				590 ± 70 BP	515–668 calBP (1,435–1,282 calAD)	
				780 ± 80 BP	563–905 calBP (1,387–1,170 calAD)	
South Africa	Balerno Shelter 3	*Achatina*	y	1,650 ± 50 BP	1,412–1,693 calBP (538–257 calAD)	van Doornum 2014
				2,270 ± 50 BP	2,153–2,353 calBP (203–403 calBC)	
	Border Cave	*	y	–	–	Beaumont 1978; *
	Bushman Rock Shelter	*Achatina*	y	9,570 ± 55 BP	10,717–11,131 calBP (8,767–9,181 calBC)	Dayet et al. 2017; Plug 1982
				12,950 ± 40 BP	15,279–15,686 calBP (13,329–13,736 calBC)	
	Castle Rock	*Achatina*	y	Iron Age	–	Calabrese 2000; Wood 2006
	Ficus Cave	*Achatina*	y	330 ± 50 BP	302–495 calBP (1,648–1,455 calAD)	Partridge 1966; Vogel and Marais 1971
	Harmony	*Achatina*	y	320 ± 25 BP	306–462 calBP (1,644–1,488 calAD)	Evers 1975, 1979
	iNkolimahashi Rock Shelter	Land snail	y	550 ± 45 BP	511–649 calBP (1,439–1,301 calAD)	Mazel 1999
	K2 (Bambandyanalo)	*Achatina*	y	Iron Age	–	Hattingh and Hall 2009; Steyn and Nienaber 2000; Wood 2006
	Leokwe Hill	*Achatina*	y	880 ± 25 BP	730–905 calBP (1,220–1,045 calAD)	Calabrese 2000; Hall and Smith 2000
				1,000 ± 60 BP	782–1,055 calBP (1,168–895 calAD)	
	Magogo	*A. immaculata*, *M. kraussi*, Achatinidae	y	1,190 ± 50 BP	981–1,258 calBP (969–692 calAD)	Maggs and Ward 1984; Ward and Maggs 1988
				1,360 ± 50 BP	1,012–1,281 calBP (938–669 calAD)	
	Mapungubwe	Snail	y	Iron Age	–	Meyer 2000
	Mhlopeni	*Metachatina*	y	1,410 ± 50 BP	1,263–1,406 calBP (687–544 calAD)	Maggs and Ward 1984
	MNR 74	*Achatina*	y	729 ± 31 BP	573–726 calBP (1,377–1,224 calAD)	Antonites et al. 2016
				837 ± 35 BP	683–895 calBP (1,267–1,055 calAD)	
	Mutamba	*Achatina*	y	Iron Age	–	Antonites 2012
	Mpambanyani	Achatinidae	y	885 ± 50 BP	705–919 calBP (1,245–1,031 calAD)	Robey 1980; Ward and Maggs 1988
				980 ± 50 BP	769–977 calBP (1,181–973 calAD)	
	Ndondondwane	*A. immaculata*, *M. kraussi*, Achatinidae	y	1,190 ± 50 BP	981–1,258 calBP (969–692 calAD	Fread 2007; Stoffberg and Loubser 1984; Ward and Maggs 1988
				1,230 ± 50 BP	1,012–1,281 calBP (938–699 calAD)	
	Ntshekane	*A. immaculata*, *M. kraussi*, Achatinidae	y	1,100 ± 50 BP	928–1,173 calBP (1,022–777 calAD)	Maggs and Michael 1976; Ward and Maggs 1988
	Ntsitsana	Shell		1,180 ± 50 BP	973–1,255 calBP (977–695 calAD)	Prins and Granger 1993
				1,290 ± 50 BP	1,082–1,299 calBP (868–651 calAD)	
	Penge	*Achatina*	y	1,318 ± 26 BP	1,184–1,295 calBP (766–655 calAD)	Antonites et al. 2014
	Pont Drift	*Achatina*	y	810 ± 50 BP	666–899 calBP (1,284–1,051 calAD)	Hanisch 1980
				1,110 ± 50	932–1,173 calBP (1,018–777 calAD)	
	Princess Hill	Shell		Iron Age	–	Antonites 2012; Loubser 1989
	Schroda	*Achatina*	y	780 ± 50 BP	657–791 calBP (1,293–1,159 calAD)	Hanisch 1980; Hall and Smith 2000
				840 ± 50 BP	677–905 calBP (1,273–1,045 calAD)	
	Tloutle	*Achatina*	y	375 ± 65 BP	305–516 calBP (1,645–1,434 calAD)	Mitchell 1993
				5,080 ± 80 BP	5,646–5,990 calBP (3,696–4,040 calBC)	
				6,140 ± 100 BP	6,759–7,261 calBP (4,809–5,311 calBC)	
				6,910 ± 80 BP	7,609–7,930 calBP (5,659–5,980 calBC)	
				8,680 ± 70 BP	9,534–9,889 calBP (7,584–7,939 calBC)	
	Verulam	*Achatina*	y	–	160–80 calBP (1,790–1,870 calAD)	Loubser 1991
					370–270 calBP (1,580–1,680 calAD)	
	Vhunyela	Shell		Iron Age	–	Antonites 2012
Tanzania	Fukuchani	Shell		Iron Age	–	Faulkner et al. 2017
	Gonja Kalimani	Land snail	y	Iron Age	–	Walz 2010a
	Gonja Maore	*Achatina*/land snail	y	–	342–468 calBP (1,608–1,482 calAD)	Soper 1967; Walz 2010a, 2010b
				1,080 ± 115 BP	769–1,265 calBP (1,181–685 calAD)	
				–	812–897 calBP (1,138–1,053 calAD)	
	Jangwani I	SNAIL	y	Pastoral Neolithic	–	Mehlman 1989
	Kilwa	SHELL		Iron Age	–	Soper 1967
	Kwa Mgogo	LAND snail (9 species)	y	–	1,102–1,234 calBP (848–716 calAD)	Walz 2010a, 2010b
					995–1,051 calBP (995–899 calAD)	
	Magubike	*	y	371 ± 23 BP	319–501 calBP (1,631–1,449 calAD)	Willoughby 2012; *
				397 ± 23 BP	332–509 calBP (1,618–1,441 calAD)	
				403 ± 23 BP	334–510 calBP (1,625–1,455 calAD)	
				1,732 ± 23 BP	1,569–1,703 calBP (381–247 calAD)	
	Mlambalasi	*	y	151 ± 24 BP	480–315 calBP (1,470–1,635 calAD)	Biittner et al. 2017; *
	Mumba	*	y	–	454–304 calBP (1,496–1,646 calAD)	Prendergast et al. 2007; *
					472–290 calBP (1,478–1,660 calAD)	
					1,922–1,615 calBP (28–335 calAD)	
	Unguja Ukuu	Shell		Iron Age	–	Chittick 1974; Faulkner et al. 2017; Wynne-Jones 2016
Zambia	Chilimulilo	Shell		Iron Age	–	Musonda 1984
	Kalemba Rock Shelter	*Achatina*/land snail	y	115 ± 70 BP	0–283 calBP (1,950–1,667 calAD)	Derricourt 1976; Phillipson 1976
				4,480 ± 90 BP	4,862–5,434 calBP (2,912–3,484 calBC)	
				5,040 ± 110 BP	5,491–6,095 calBP (3,541–4,145 calBC)	
				7,030 ± 105 BP	7,657–8,037 calBP (5,707–6,087 calBC)	
	Makwe	*Achatina*/land snail	y	4,380 ± 130 BP	4,585–5,440 calBP (2,635–3,490 calBC)	Phillipson 1976; Yamasaki et al. 1972
				4,920 ± 130 BP	5,326–5,922 calBP (3,376–3,972 calBC)	
	Mufulwe	Shell		Iron Age	–	Gutin and Musonda 1985; Musonda 1984
	Mwambacimo	Shell	y	–	–	Musonda 1984
	Nachikufu Shelter	Snail/shell	y	1,060 ± 100 BP	747–1,232 calBP (1,203–718 calAD)	Miller 1969
	Nsalu Hill Cave	Snail	y	Later Stone Age	–	Miller 1969
	Thandwe Rock Shelter	*Achatina*/land snail	y	890 ± 110 BP	656–1,050 calBP (1,294–900 calAD)	Phillipson 1976; Yamasaki et al. 1972
Zimbabwe	Chiwona Kopje	Shell		Iron Age	–	Caton-Thompson 1931
	Danamombe	*Achatina*	y	Iron Age	–	Machiridza 2012
	Great Zimbabwe	*Achatina*/shell	y	Iron Age	–	Beck 1931; Caton-Thompson 1931
	Hlamba Mlonga Hill	*Achatina*	y	720 ± 50 BP	558–735 calBP (1,392–1,215 calAD)	Thorp 2009
				780 ± 50 BP	657–791 calBP (1,293–1,159 calAD)	
				1,040 ± 40 BP	832–1,057 calBP (1,118–893 calAD)	
	Hubvumi Ruins	Shell		Iron Age	–	Caton-Thompson 1931
	Kadzi River	*Achatina*	y	990 ± 50 BP	785–1,045 calBP (1,165–905 calAD)	Plug 1997
				1,290 ± 50 BP	1,082–1,299 calBP (868–651 calAD)	
	Khami	Shell		290 ± 30 BP	288–457 calBP (1,662–1,493 calAD)	Mukwende 2016
				350 ± 30 BP	315–492 calBP (1,635–1,458 calAD)	
				430 ± 30 BP	335–529 calBP (1,615–1,421 calAD)	
	Malumba	*Achatina*	y	690 ± 95 BP	517–792 calBP (1,433–1,158 calAD)	Bvocho 2005; Manyanga 2006; Manyanga et al. 2000
				965 ± 80 BP	707–1,053 calBP (1,243–897 calAD)	
	Matendere	Shell		Iron Age	–	Caton-Thompson 1931
Zimbabwe	Mapela	Shell		770 ± 30BP	669–733 calBP (1,281–1,217 calAD)	Chirikure et al. 2014; House 2016
				900 ± 30 BP	740–911 calBP (1,210–1,039 calAD)	
	Mtao Village 16	*Achatina*/shell	y	420 ± 45 BP	319–532 calBP (1,631–1,418 calAD)	Manyanga 2006
	Mutshilachokwe	Shell		910 ± 60 BP	705–931 calBP (1,245–1,019 calAD)	Manyanga 2006
				670 ± 60 BP	540–696 calBP (1,410–1,254 calAD)	
	Mwenezi	*Achatina*	y	1,250 ± 75 BP	987–1,298 calBP (963–652 calAD)	Bvocho 2005; Manyanga 2006; Manyanga et al. 2000
				800 ± 70 BP	653–910 calBP (1,297–1,040 calAD)	
	Samakande	*Achatina*	y	1370 ± 35 BP	1,188–1,348 calBP (762–602 calAD)	Manyanga and Shenjere 2012
				1,580 ± 35 BP	1,396–1,546 calBP (554–404 calAD)	
	Tshobwane	Shell		790 ± 60 BP	572–902 calBP (1,378–1,048 calAD)	Manyanga 2006
				890 ± 60 BP	700–922 calBP (1,250–1,028 calAD)	

The asterisk refers to this publication as the original reporting

Use of variable terminology presented the greatest challenge to this literature review. Without an established convention to identify or report LSS beads, numerous published instances may or may not refer to the same raw material. Sites where the shell bead material was identified as OES, marine shell, or water snail were excluded from this list. Some sites have reference to “shell beads”; however, further searching reveals their identification as something other than land snail, e.g., “small ocean snails” at Takwa (Mukhwana 1992, p. 20), “marine gastropod” of likely *Andara* sp. at Manda (Mann 2000, p. 37), and “fresh water mollusc” at Gamble’s Cave (Wandibba 1988, p. 20), all from Kenya. The publications which used the generic term “shell beads” were included in our list, as further study is necessary to determine what type of shell they may be.

To distinguish between potential LSS cases, the raw material category in Table 1 has two sub-columns to highlight the different standards in reporting. The first lists the term used in the original publication cited. By far, the most common term in the published literature is *Achatina*, but this is one genus among hundreds of identified species of African land snails (Tattersfield 1998). Some site reports have narrower classifications to a particular species, e.g., *A. immaculata* at Ntshekane, South Africa (Ward and Maggs 1988). Others use the more generic term “mollusc shell,” such as North Horr 1, Kenya (Wandibba 1988). Based on this information, the second sub-column indicates whether the case is likely to be an LSS bead. Beads which are not indicated as LSS may still belong to this category; however, more information is required to support such a designation. These cases still warrant inclusion in our table as they could represent land snails and merit further study.

Radiocarbon dates and ages provided in Table 1 are based on published dates and archaeological contexts. We calibrated all published radiocarbon dates using Intcal13 and reported to 95% confidence interval or 2*σ* (Reimer et al. 2013). Unfortunately, chronological control for such finds is often lacking. Direct bead dates would be the most reliable given the stratigraphic mobility of beads; however, no direct LSS dates are available in the existing literature. The next most desirable option is to report dates from an associated layer. Where undated, we report the date from the next closest appropriate context (i.e., the underlying or overlying strata). Many publications report shell beads only as present/absent, or as an overall number from the excavation. In cases without provenience and/or associated dates, we report bracketing dates for the site or a broad time period (Later Stone Age, Pastoral Neolithic, or Iron Age).

## Background on African Land Snails

Investigating the phenomenon of LSS beads first requires some background on the raw material. Shelled gastropods, commonly referred to as snails, live in a variety of terrestrial and aquatic environments. Lunged, air-breathing gastropod species belong to the informal group Pulmonata (Cuvier in Blainville 1814) and are primarily terrestrial (Bouchet and Rocroi 2005). Three major tropical snail families within the achatinoid (Stylommatophora) clade are prevalent throughout sub-Saharan Africa (Rowson et al. 2011). These include the carnivorous hunter snail family Streptaxoidea (Gray 1860), the awl snails of Subulinidae (Fischer and Crosse 1877), and the giant African land snail family, Achatinidae (Swainson 1840). There are approximately 254 species within the Achatinidae family. Eight Achatinidae genera have taxa with the shell length, shapes, and thickness necessary to produce a dense, non-curved blank for beadmaking. Subulinidae and Streptaxoidea shell lengths range from < 1 to ~ 30 mm and have depressed trochiform shapes which are too thin and curved for disc bead production.

**Fig. 2:**
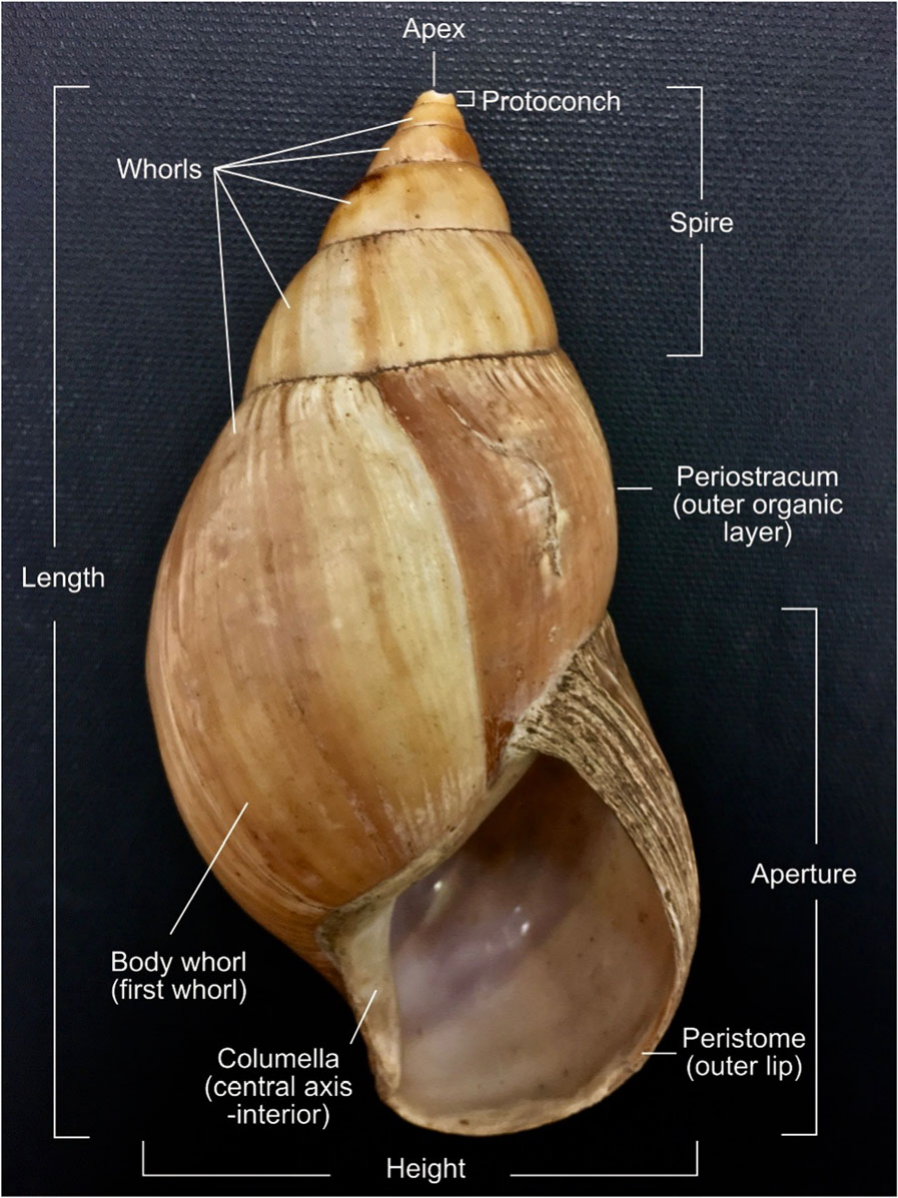
Annotated diagram of terms used to describe gastropod shells (images in full color online)

A snail’s shell (Fig. 2) encloses the soft parts of the body creating support for the animal and protection from the environment, predators, and dehydration (Avery and Etter 2006; Goodfriend 1986; Vermeij 1978). Land snails begin growing from a thin, soft protoconch shell by depositing calcium carbonate and conchiolin from the mantle tissue at the peristome (outer lip) of the shell aperture, or opening (Wilbur and Saleuddin 1983). This forms the apex of the shell spire and gradually coils forward to produce the whorls and first body during the snail’s ontogeny. In most land snails, the outer lip of the shell becomes reflected and the shell stops growing once the animal reaches sexual maturation (Goodfriend 1986; Wolda 1970). However, *Achatina* (also known as *Lissachatina fulica* [Bowdich 1822]), the most invasive giant African land snail currently in eastern Africa, does not develop a reflected lip at adulthood. Instead, they continue shell growth and calcium carbonate deposition to produce a thickened peristome (rim around the opening) despite sexual maturation (Tomiyama 1993). The mean peristome thickness observed in old adults is > 0.8 mm compared to < 0.5 mm in young adults (Tomiyama 1993).

Achatinidae are remarkably resilient and can live in a number of environments and conditions. They are nocturnal, herbivorous creatures who can forage on up to 500 different types of plant species, making them extremely adaptable to varied rainforest and savannah environments (Bhattacharyya et al. 2014; Chukwuka et al. 2014). These snails prefer warm and moist conditions and are drawn to ephemeral water sources, although they live entirely on land. During less favorable hot and dry conditions, Achatinidae enter a state of dormancy (aestivation) which can last from 4 weeks to over a year, although longer durations increase risk of mortality (Rees and Hand 1993). During aestivation, snails enter a light state of dormancy with reduced activity and a lowered metabolic rate. They accomplish this by secreting an epiphragm (a calcareous mucus plug) that blocks the aperture and allows the animal to attach to a substrate, or in some cases, other snails during these adverse environmental conditions (Salway et al. 2010). This physiological state can be rapidly reversed when conditions become hospitable again. All pulmonate species are hermaphroditic once they reach old adulthood—meaning they produce both sperm and eggs. Individuals can start breeding around 6–8 months of age and can breed multiple times a year, producing clutches of between 30 and 1,000 eggs (Bhattacharyya et al. 2014; Tomiyama 1993).

Several components of shell morphology can be used to distinguish between gastropod species. One is the aperture ontogeny, which includes both the height and diameter of the aperture, as well as the growth trajectory. For example, *A. fulica* has a decreased width and longer height, which produces a long, narrow conical-shaped shell. By contrast, *A. glutinosa* (Pfeiffer 1854) has a more equitable aperture height and length, which produces a shorter and more bulbous shell. The number of whorls and the chirality, or direction of coiling, are also informative. Gastropods have between 2 and 10 whorls within the spire, with a higher number imparting greater mechanical strength and an elongated form (Rice 1998). Chirality can be either dextral (right handed) or sinistral (left handed) (Gittenberger et al. 2012). Dextral spiraling accounts for 90% of all organisms, with all members of the same species taking the same form.

It is sometimes possible to tell different taxa apart based on the colors and patterns of the periostracum and prismatic layers (Wrigley 1948, but also see Owen and Reid 1986). The periostracum is a thin layer of sclerotized protein conchiolin that covers and protects the entire shell and enhances its coloration (Watabe 1988). For example, *Achatina achatina* (Linnaeus 1758) is well-known for its tiger-striped appearance. However, this is a less reliable means of species identification. First, the colors can vary with habitat, causing members of the same species to appear different depending on local conditions. Secondly, the periostracum erodes soon after the death of the organism (Watabe 1988). Although the rate of disintegration can be slowed by calcareous and/or anaerobic environments, it is commonly destroyed within 1 year postmortem. Other identifications are based on the coloration of the outer lip, parietal wall, and columella (coiling axis). The parietal wall and columella of *A. achatina* is reddish-wine color and the outer lip is blueish white (Bequaert 1950). On the other hand, *A. tögoensis* (Bequaert and Clench 1934) exhibits a blueish white hue for all three structures while maintaining the tiger-striped outer shell (Bequaert 1950). In general, archaeological snail taxa cannot be determined using color because of the loss of the periostracum, as well as the tendency for shells within deposits to be fragmented either naturally or potentially through human activities and taphonomically altered (e.g., by oxidized ferric soils, sun-bleaching). Therefore, in archaeological contexts, shell morphology and morphometrics are better means of taxonomic identification.

Geographic distributions of Achatinidae are based on observations from the past 200 years, which are unlikely to provide a reliable proxy for ancient distributions. At present, eastern Africa is dominated by *Achatina* (*Lissachatina*) *fulica* (Bowdich 1822), *A. immaculata* (Lamarck 1822), *A. albopicta* (Smith 1878), and various subspecies within these taxa (Verdcourt 1983; Williams 1951). Conversely, species found in southern Africa include members of the *Achatina* genera, as well as several varieties of *Archachatina* sp., which are distinguishable by a blunter spire and more globose body form than *A. fulica*. Western and central Africa are inhabited by still different species (Awodiran et al. 2015; Bequaert and Clench 1934; Chukwuka et al. 2014) such as *A. achatina* (Linnaeus 1758), *A. rugose* (Putzeys 1898), and *A. balteata* (Reeve 1849). Present day species distributions likely do not reflect prehistoric conditions because of the effects of modern transportation, environmental change, farming activity, and deforestation, as well as other aspects of human impact. More localized research is needed to reconstruct Pleistocene and early Holocene Achatinidae and other land snail ecologies. Archaeological research on snail distributions, however, must contend with biases surrounding how snails infiltrate deposits.

Snails enter the archaeological record in several ways. One is through their own behavior in life, including burrowing into deposits for the purposes of aestivation. Snails are a known bioturbation agent, burrowing down between 5 and 1 m depending on species and localized conditions, although they tend to cluster just beneath littoral debris on the ground surface. The presence of snail shell can also reflect predation from animals (e.g., mongoose, rodents, land crabs, or carnivorous land snails) or humans (Marlowe 2010; Mead 1961; Walz 2017; Williams 1951). Whereas predation by the former group tends to leave small clusters of shell and/or telltale puncture marks (Walz 2017, p. 92), human snail-harvesting activities are more ambiguous. It has been suggested that sites with high densities of snail shells must reflect human harvesting activities, e.g., at Mumba, Tanzania (Mehlman 1979, pp. 87–88) and Kuumbi Cave, Zanzibar (Shipton et al. 2016). Further evidence for purposeful snail harvesting can be found in death profiles in which all the snails were fully mature and of limited type, as opposed to a natural death profile with greater variation in size, age, and species (Evans 1972). Narrow size ranges for collected specimens and breakage around the aperture and just above the body whorl for putative meat extraction may also support the hypothesis of human accumulation (Shipton et al. 2016, pp. 216–218). These indicators are well established in the context of Capsian *escargotières* found throughout the North African Maghreb between 10,000 and 6,000 BP (Lubell 2004; Lubell et al. 1976) and have also been used to argue for aquatic resource exploitation in the Horn of Africa (Kappelman et al. 2014).

However, it is worth noting that evidence for snail harvesting south of the Sahara is significantly patchier (summary in Mehlman 1979, pp. 87–88). Proposed archaeological cases are almost entirely restricted to later Pleistocene contexts, perhaps related to the diversification of diets and the “Broad Spectrum Revolution” (Binford 1968; Flannery 1969; Stiner 2001). Contemporary snail eating among African communities is also well documented (e.g., Marlowe 2010; Mead 1961; Walz 2017). It is therefore curious that Holocene archaeological cases are rare. Harrison and Mbago (1997) report the sole case at a potential Pastoral Neolithic site in Tanzania. Otherwise, snail consumption is sometimes offered as an ad hoc explanation for how shell arrived at LSS bead sites—e.g., at Bushman Rockshelter (Plug 1982), Tloutle (Mitchell 1993), and Mumba (Mehlman 1989)—although there is little supporting evidence in the form of middens.

Importantly, the normative behavior of snails also contributes to the formation of high-density assemblages upon which most subsistence arguments are based. Snails tend to congregate in favorable patches, e.g., around crops or water sources. The relative abundance of snail species can therefore be used to reconstruct the local environmental conditions, specifically the availability of freshwater. For example, Walz (2010a, p. 211) links an increase in unbroken amphibious snail shells to the onset of a wetter phase at Kwa Mgogo, Tanzania. However, rapid changes in localized conditions can create catastrophic death profiles, wherein dozens of snails die within these highly localized clusters (Evans 1972). The presence of many snails in one layer can therefore reflect natural processes as easily as human harvesting activities. Distinguishing between these possibilities requires careful attention to the quality of the shell assemblages beyond subjective measures of density and unfortunately may not be possible in many cases.

Ultimately, land snails are common ecofacts of sub-Saharan African archaeological sites and whether or not their presence reflects human subsistence does not impact their availability as a raw material. The sheer number of sites with LSS beads underscores the pervasiveness of snail shell use in material culture, reiterating the need for greater attention beyond the dichotomy of  “natural” or “subsistence” (Walz 2017). We now turn to the practical task of determining when disc beads are manufactured from land snails. Although identifying species is difficult in fragmented shell, and likely impossible with worked shell where the outer layers have been completely removed, it may still be possible in many situations to distinguish land snail shell from other options.

## Identifying Snail Shell as a Raw Material

Identifying small, worked shell beads to taxa can be challenging. Ostrich eggshell is one of the most recognizable materials because of its frequency at sub-Saharan archaeological sites and the regular dotted patterns of pores on the cuticle surface. Non-OES shell and worn beads of various materials can be more difficult to identify. In early production stages, mollusc beads are easily distinguished from OES by the shiny nacre on their inner surface and details on the outer surface such as distinctive ridges or colored patterns. However, on heavily manufactured or worn beads, these features tend to be replaced by a smooth, unremarkable surface. As a result, worn beads made from OES, LSS, marine shell, bone, or ivory may appear superficially similar.

Further problems arise when trying to identify LSS beads to species. Taxonomic identification of snails depends on the size and morphology of key features such as the aperture and spire that are typically absent on disc beads which are produced from the first body whorl. Although most cases are reported as *Achatina*, even confidence in genus-level identification is questionable given the removal of identifying features during bead production, fragmentary nature of most shell assemblages, paucity of information on ancient species distributions, and a lack of comparative collections.

Unmodified snail shells in a site’s deposits may be helpful in determining the kind of raw materials available for bead manufacture; however, this must be done with several caveats. As discussed, snails burrow into deposits during life, so they may not be contemporary with the archaeological stratum. Secondly, species that are abundant in the area may not represent the best shell for bead manufacture. If there is no evidence of bead manufacture on site, such as incomplete beads or blanks, it is also possible that the species diversity within the assemblage is not the same as that where the beads were produced. Therefore, although unmodified shells may rule in or out potential taxa for raw material, they are not sufficient to identify bead raw material to species.

Distinguishing LSS beads in the absence of visible shell traits therefore rests on the ability to assign the shell to the more general category of land snail, which can be done in most cases using surface morphology. All molluscs have at least three types of structural layers—periostracum, prismatic, and homogenous—with some having a fourth cross-lamellar layer (Claassen 1998). Ward and Maggs (1988) were the first to suggest that this microstructure can be used for identification in sectioned fragments of LSS as well as beads. They note that the outer surface of *Achatina*, if sufficiently preserved, exhibits irregular ridging in parallel rows that are frequently yellowish to pinky-brown. The laminae also tend to have a low diagonal angle reflective of the growth pattern of the shell. They report other potential Achatinidae shells as showing a characteristic ripple pattern and laminated structure. However, they caution that these features are unreliable for identification purposes if the bead lacks clear outer surface features of the shell (Ward and Maggs 1988, pp. 410–411). This is nevertheless an intriguing idea that bears further consideration. The ability to identify this raw material from bead side profiles under low magnification would constitute a simple and non-destructive method for recognizing LSS beads and distinguishing them from OES, regardless of wear. To further investigate the utility of bead cross-sectional morphology, we turn to a new case study from southern Tanzania.

### Case Study: LSS Beads from Magubike Rockshelter, Tanzania

Magubike Rockshelter (HxJf-01) is located in the southern highlands of Iringa and possesses a stratified sequence of Middle Stone Age (MSA) to recent Iron Age/Historic deposits (Werner and Willoughby 2017; Willoughby 2012). The site possesses some of the oldest OES beads in sub-Saharan Africa, directly dated at (and beyond) the radiocarbon limit (Miller and Willoughby 2014). Both OES and non-OES shell beads are present in the deposits; however, the latter are only found in the uppermost levels. Glass beads are also present in the historic levels. Here, we report 61 potential LSS beads recovered from different parts of the rockshelter.

Potential LSS beads at Magubike were recovered in two different areas: the central excavation block (*n* = 58) and toward the rear of the shelter (*n* = 3). Those excavated from the central block in 2012 were found between 10 and 50 cm below the modern ground surface. When considering the entire collection from the central excavation, average bead diameter is 6.35 mm (4.87–7.81 mm range), with an average thickness of 1.49 mm and aperture diameter of 2.05 mm. It seems plausible that these beads were deposited around the same time and may belong to a single feature. None were in early stages of production, suggesting import to the rockshelter in a complete state. This bead concentration was associated with lithics, ceramic sherds, and iron slag. The remaining beads were excavated in 2016 toward the back of the rockshelter from a depth of approximately 25–30 cm below surface. Although there are only three beads from this area, and one is broken, they appear different from those in the main excavation area. These beads are significantly smaller in diameter (4.23 mm), with two of them having a peculiar squared shape.

**Fig. 3:**
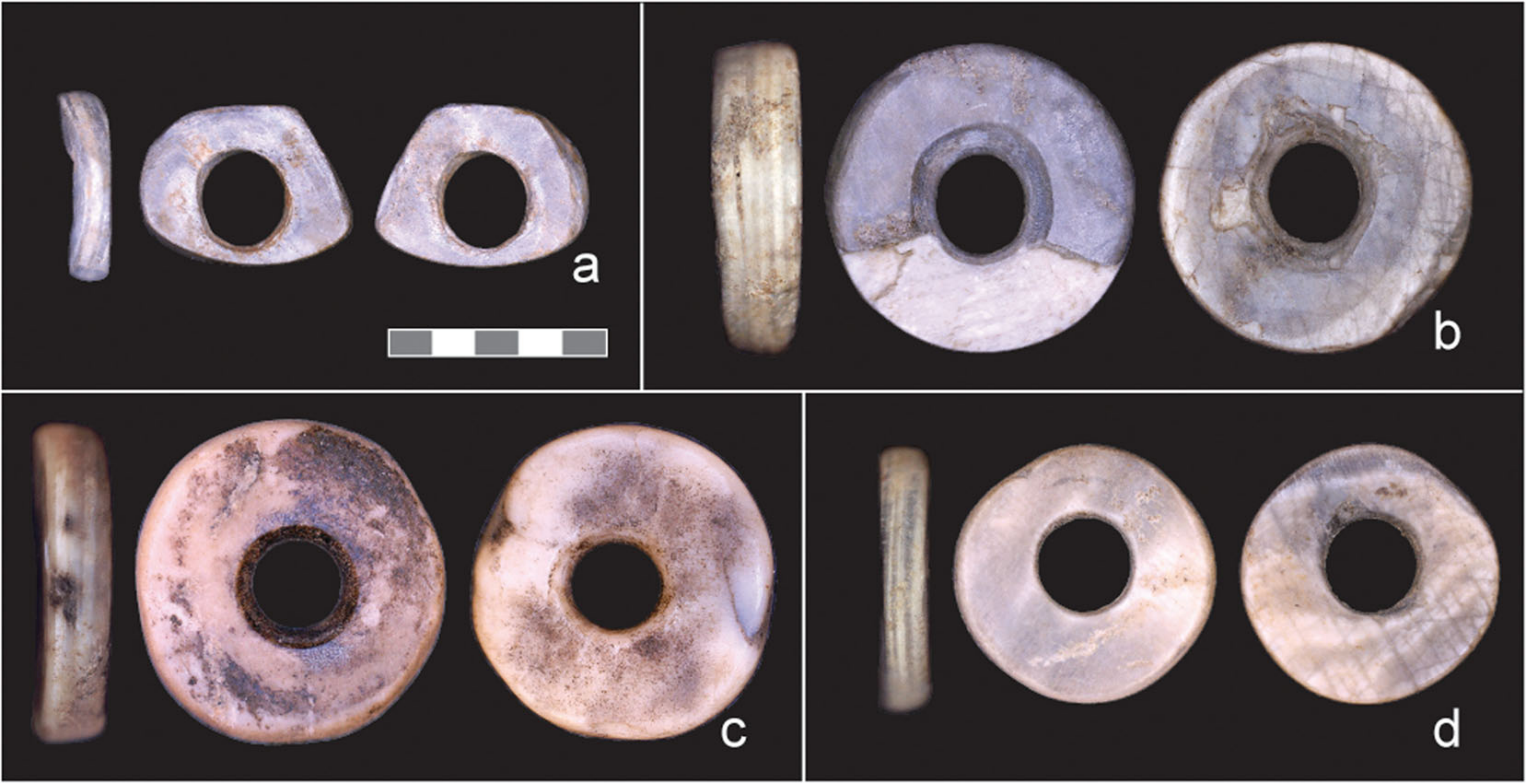
Directly AMS dated potential LSS beads from Magubike. **a** Radiocarbon sample UOC-4739. **b** UOC-4742. **c** UOC-4741. **d** UOC-4740 (photos: by authors)

In order to better understand the chronology bead use at Magubike, we submitted four potential LSS beads for radiocarbon dating: three from the central bead concentration and one from the back of the shelter (Fig. 3, Table 2). There are known problems with radiocarbon dating of land snail shells, notably the potential for molluscs to incorporate old ^14^C from marine environments or bedrock during shell production (i.e., the reservoir effect) (see Goodfriend and Hood 1983; Wright 2017; Xu et al. 2011). However, affected terrestrial environments tend to have limestone or shale bedrock, and the Iringa region is dominated by metamorphic quartzites and granites (Biittner et al. 2017). Ultimately, snail shells are commonly dated in African contexts when other appropriate organic material is scarce. The three beads from the central unit have similar dates ranging from 1,455 to 1,632 calAD. This is broadly consistent with them being part of a single depositional feature dating to the later Iron Age. The bead toward the rear of the shelter, which differs in size and morphology, is significantly earlier: 327–414 calAD. Either old shells were gathered and used for bead production, or the archaeological deposits at Magubike reflect at least 1,000 years of potential snail shell bead use in southern Tanzania. Although we cannot yet distinguish between these possibilities, vast differences in the size and morphology of beads from the two areas lend support for distinct occupations.

**Table 2:** Direct AMS radiocarbon dates of four potential LSS beads from Magubike Rockshelter

Lab ID	Provenience	^14^C yr BP	95% CI calBP
UOC-4739	MAB06 level 5 SE	1732 ± 23	1,700–1,536
UOC-4740	TP8 20–30 cm	403 ± 23	496–326
UOC-4741	TP8 20–30 cm	371 ± 23	470–318
UOC-4742	TP8 30–40 cm	397 ± 23	494–325

Calibration performed using OxCal v4.2.4 (Bronk Ramsey 2009) and the IntCal13 calibration curve (Reimer et al. 2013)

#### Distinguishing LSS from Other Shell

To determine shell raw material, we compared the potential LSS beads at Magubike with modern and unmodified archaeological shells from the study region. Mollusc taxa can be identified using several visible characteristics, including shell color, pattern, and texture. However, when these obvious macroscopic traits are eroded or absent, the microstructure of the shell wall can distinguish LSS from other types of molluscs (Ward and Maggs 1988). High-power microscopy is not required; a 10×–20×-magnification hand lens or a handheld digital microscope is sufficient to see the relevant cross-sectional attributes.

Following Ward and Maggs (1988), we compared the bead side profiles with sectioned marine and land snail shells to examine visible microstructure under low magnification. The 61 Magubike beads assessed have distinctive stacked laminations in profile (Fig. 5a–c). Disregarding one broken and delaminated specimen, the average maximum thickness of these beads is 1.48 mm, with a range from 0.92 to 2.17 mm. None have retained the sculptural details from the prismatic layer, suggesting the finished bead form is thinner than the original shell. Absence of the prismatic layer supports the idea that bead production/wear is capable of removing the identifiable, superficial characteristics of the outer shell.

**Fig. 4:**
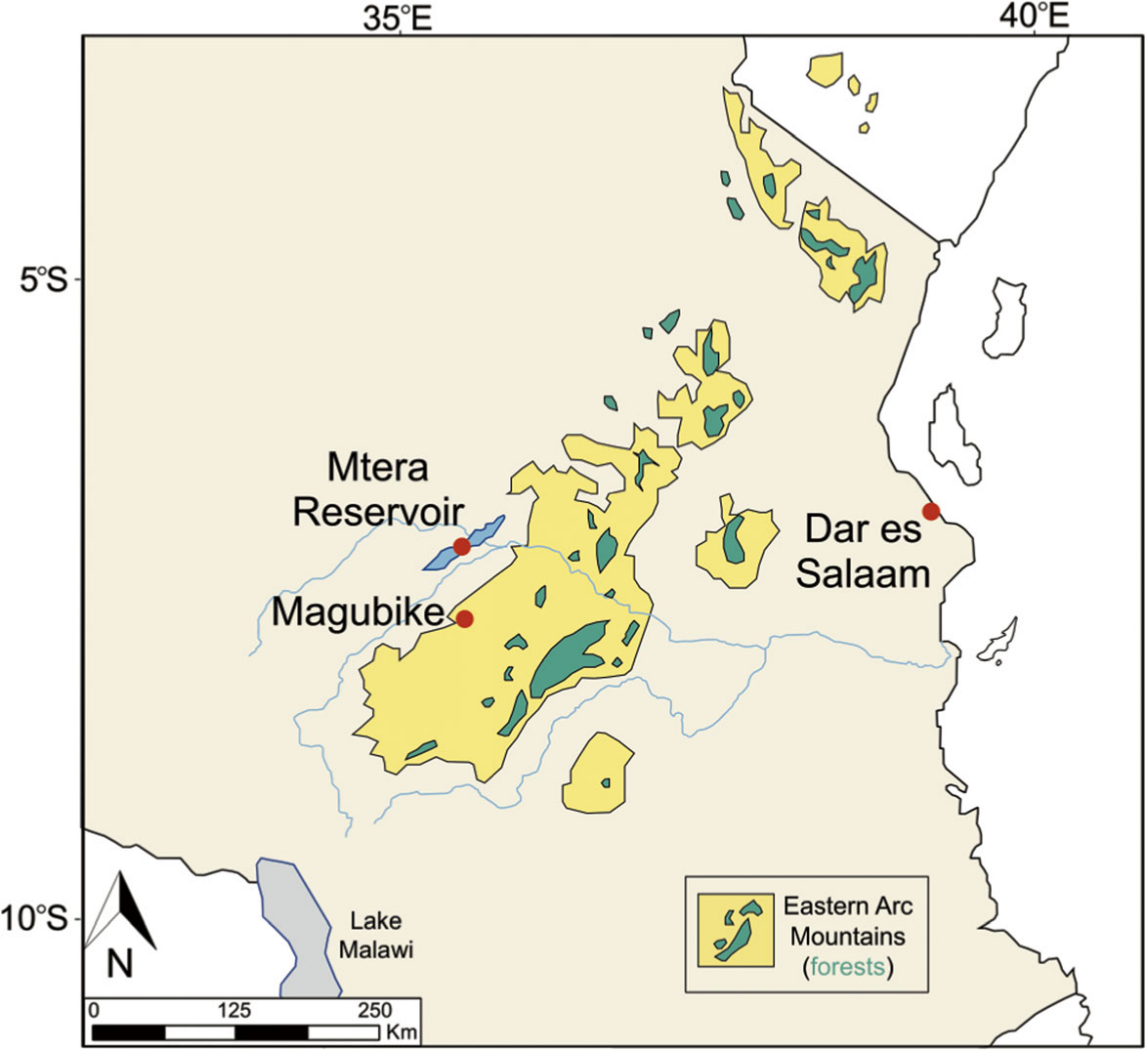
Relative locations of shell samples collected for analysis, in Tanzania

**Fig. 5:**
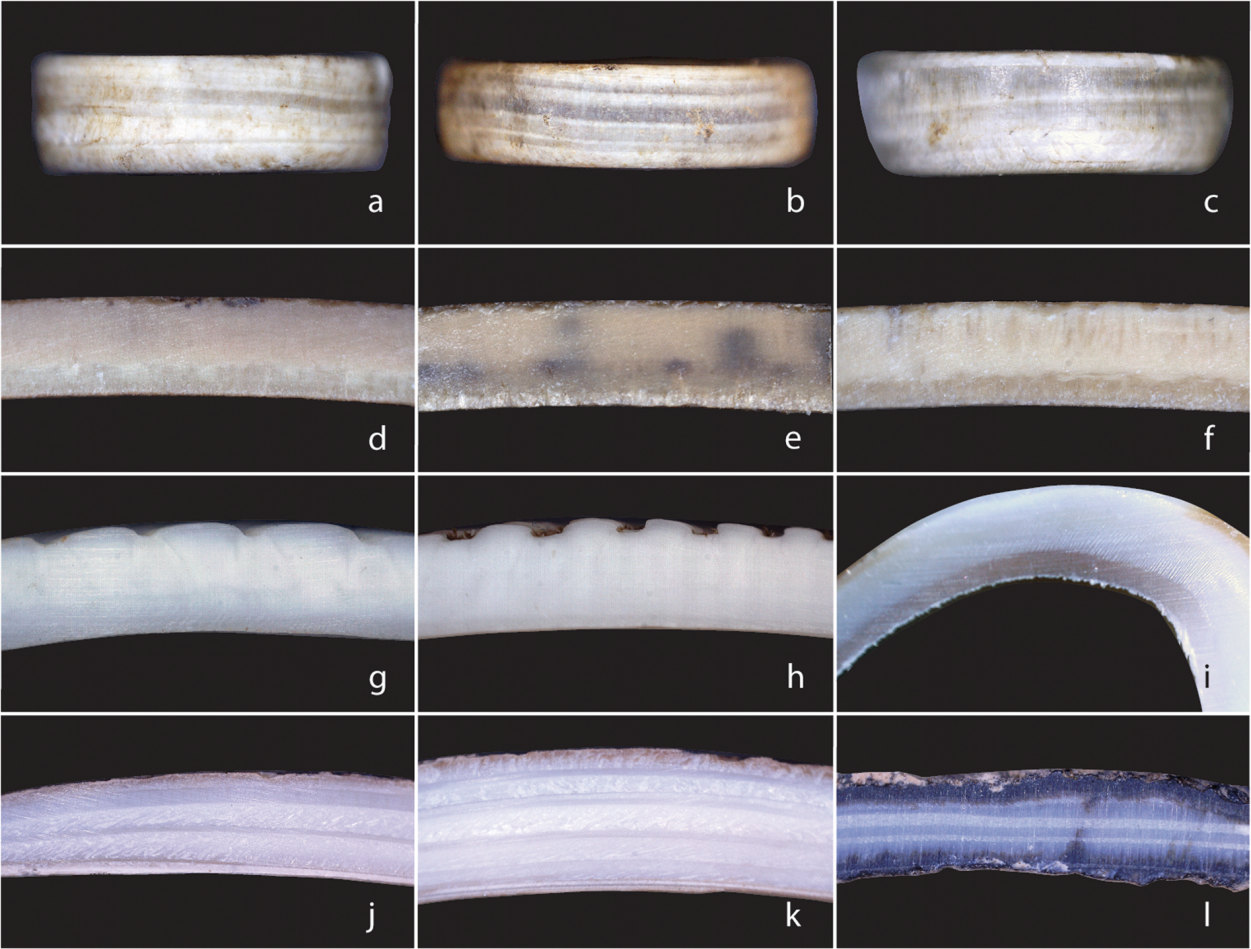
Comparative shell microstructure. **a**–**c** Beads from Magubike. **d**–**f** OES from Magubike. **g**–**i** Marine shell from Dar es Salaam. **j**–**l** LSS from Magubike (photos: by authors)

Comparative shell samples were collected from the Tanzanian coast, Mtera Reservoir, and the archaeological deposits at Magubike (Figs. 4 and 5). Sections were ground using a Buehler Ecomet III Polisher/Grinder at the University of Alberta. These cross sections, along with thickness measurements and published descriptions of shell microstructure, form the basis for our attribution of the Magubike beads to the land snail category.

The first shell taxon to be considered and ruled out as potential beadmaking material is OES (Fig. 5d–f). Samples of this raw material were taken from the main excavation block at Magubike, from between 10 and 40 cm below surface. All OES from the site is artifactual; however, these three samples were from very early bead production stages and as such were not decreased in thickness by use-wear. Thickness ranged from 1.82–2.30 mm and therefore could overlap with the bead material thickness discussed here. Upon viewing the microstructure of the OES, however, it becomes clear that the Magubike beads are not a match. OES has three distinct layers, the thickest of which is the palisade, with the mammillary cones visible on the inner surface (Dauphin et al. 2006; Li-Chan and Kim 2008). The cuticle layer thickness is measured in microns and will barely be visible in profile. The palisade layer has a spongy looking texture, while the mammillary layer looks like a series of tiny tubes running perpendicular to the shell’s surface (Ward and Maggs 1988, p. 408). This is a clear mismatch with the banded layers visible in the profile of the Magubike beads.

When considering non-OES possibilities, one must first distinguish between terrestrial/freshwater gastropods and marine molluscs. In general, marine shell layers appear more homogeneous in cross section when compared to the laminar stacked appearance of terrestrial and freshwater gastropods (Watabe 1988). Marine shell samples were collected from the shore at Msasani Bay, Dar es Salaam, Tanzania. Only three of the shells had sufficient thickness and shape for beadmaking, so those (Fig. 5g–i) were chosen for sectioning. Samples g and h are from unidentified bivalves, while sample i is from a gastropod, likely *Semicassis bisculata* (Schubert and Wagner 1829). All have the shiny nacre layer typical of marine molluscs and appear relatively homogenous in cross section.

The modern marine samples have some visible layers in cross section; however, their appearance is not consistent with the profile of the shell beads from Magubike. Although there are some visible layers, the inner structure appears relatively homogenous. The inner layer is most distinct, with the others appearing to blend into one another. Of the three marine samples, the gastropod (sample i) has the most apparent distinction in its layers, making it a potential candidate for the Magubike bead material; however, its pinky hue and highly coiled shell render it a mismatch. The gastropod shell is also the thinnest of the marine shells considered, measuring only 0.83 mm, while the bivalves ranged from 1.80 to 2.26 mm. Although the marine bivalves have the appropriate thickness and coloring to be potential candidates for the Magubike bead material, the distinctive laminar microstructure is absent, ruling them out as a source material.

Gastropod shells all form using the same process so have similar microstructures; however, terrestrial and freshwater snails can be distinguished based on shell size and thickness. Snail shell growth is accretionary, beginning at the outer lip of the aperture, with cross-lamellar structures forming in alternating orientations that give strength to the shell structure (Dauphin and Denis 2000; Watabe 1988). These cross-lamellar structures also give a telltale banded appearance in cross section. The distinction between freshwater and terrestrial gastropods is evident in their shell size and thickness. Mature freshwater snails tend to be smaller (and thinner) than their terrestrial counterparts due to a difference in the available environmental calcium. Land snails retrieve their calcium from numerous environmental sources such as plants, rocks, dirt, and other snails (DeWitt et al. 2000). Calcium is less available to freshwater snails, so their shells are adapted to be general thinner and smaller (DeWitt et al. 2000).

Modern aquatic snail shells were collected from the closest permanent source of freshwater to Magubike: Mtera Reservoir. A hydroelectric dam positioned where the eastern outflow of the reservoir meets the mouth of the Great Ruaha River undoubtedly disrupts the current ecosystem, but at more than 600 km^2^ in size, this naturally occurring lake would have been a good resource for Iron Age and Later Stone Age people. None of the modern freshwater snails from Mtera were of sufficient thickness to have produced beads; hence none were sectioned for comparison in Fig. 4. The largest adult specimen collected (*Bulinus* sp. Müller 1781) measured 66 mm long by 45 mm high. The thickest part of this shell, the outer lip, measured 0.60 mm. This measurement includes the prismatic layer, which is not present on any of the Magubike beads, and its removal would reduce the thickness even further. The thinnest bead from Magubike is 0.92 mm, effectively ruling out freshwater shells as potential raw material.

Comparative samples of land snail shell from Magubike are ecofacts taken from the main excavation block under the shelter overhang. The sectioned shells (Fig. 5j, k) were collected from 30 to 40 cm below surface, while sample l was recovered from 130 to 140 cm below surface. The significant differences in depth explain the dark coloring of sample l, as it is partially fossilized; however, the microstructure of the layers remains consistent and distinct from marine, freshwater, and ostrich eggshell. The outer shell layers are still present on the LSS samples rendering them slightly thicker than the resulting beads would be. The thicknesses of the samples range from 1.36 to 4.16 mm, which would be adequate to produce the archaeological beads. The distinctive laminated appearance of the inner layers is unique among the sectioned samples. While only 2–4 layers were visually distinct in the marine and OES samples, there are significantly more layers present in the LSS sections. The alternating cross-lamellar layers give a characteristic striped pattern to the microstructure that remains in the profile of the Magubike beads. Even when broken or heavily worn, the perpendicular orientation of the crystal layers of LSS have a distinctive texture of “torn plywood” (Ward and Maggs 1988, p. 411). From this information, it is possible to identify a shell bead as LSS even if the outer shell characteristics have been removed.

Examining the side profile of a disc bead under low magnification is a quick, non-destructive means of identifying shell raw material when surface characteristics are ambiguous. Our results are summarized in Table 3 for quick reference. This works builds on Ward and Maggs (1988), who suggested these microstructure patterns in their study of OES, LSS, and freshwater bivalves from the KwaZulu-Natal Province of South Africa. Although all gastropod shell formation should follow the same principles and therefore have a similar structure, we advise caution when using shell thickness as a distinguishing feature. Aquatic snails from the Mtera Reservoir were ruled out from our study based on their thin shells, but it remains unclear whether snails from a larger lake (such as Lake Malawi) could have thicker shells suitable for beadmaking. Based on our observations, land snail shells do appear to vary in thickness across Africa. Therefore, we advise a thorough examination of the locally available shell when trying to distinguish bead raw material.

**Table 3:** Summary of comparative shell microstructure, from Tanzanian samples

Shell type	Layers	Textures	Features
OES	• 3 distinct layers, palisade is the thickest	• Palisade layer appears spongy	• Mammillary layer looks like vertical tubes
Marine	• Some visible layering	• Layers look smooth, homogenous	• Inner layer is well defined
LSS	• 2 or more banded layers, depending on thickness	• Alternating rough and smooth textures	• Distinct horizontal layers, like stripes

#### What Kind of Land Snail?

As with many LSS beads, it is not possible to identify species in the Magubike case because taxonomically informative features from the outer surface of the shell were destroyed during production and use-wear. Generating a range of potential species rests in part on determining which terrestrial land snails are present in the archaeological deposits. However, this presents a secondary problem. The only complete shells at Magubike are present in lower MSA levels of the sequence, where there are no snail shell beads. Conversely, upper levels with LSS beads do not possess any complete shells that are large enough to be considered a potential raw material. This is further complicated by the behavioral processes of snails discussed earlier which affect vertical mobility of these organisms in the deposits.

**Fig. 6:**
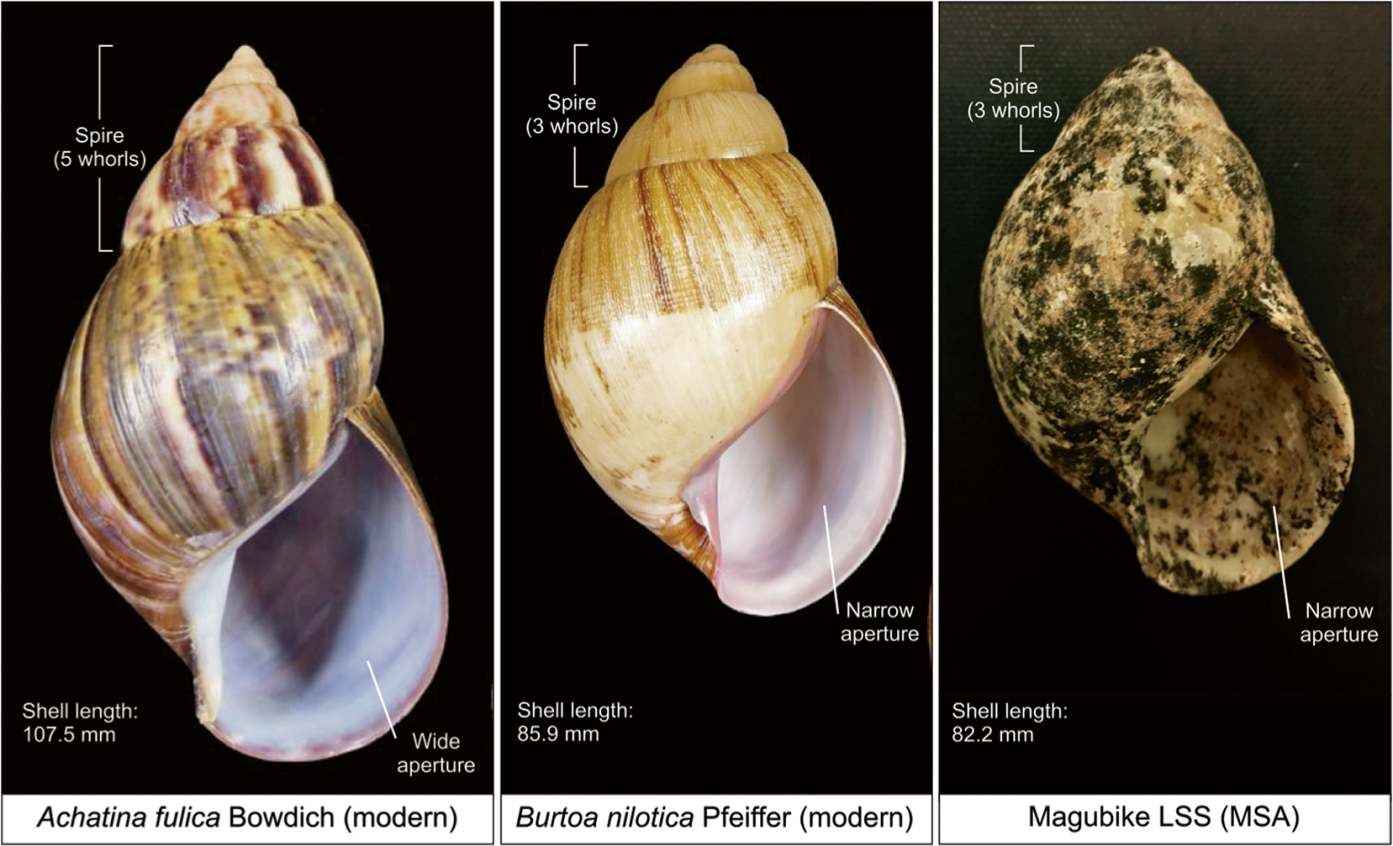
Comparison of *Achatina fulica*, *Burtoa nilotica*, and archaeological LSS from Magubike; images of *A. fulica and B. nilotica* used with permission from ©Guido & Philippe Poppe (righthand photo: by authors)

The dearth of information on past snail distributions remain a major limiting factor. Comparison of the complete MSA shells at Magubike to the region’s current endemic species—*A. fulica* (Rowson et al. 2011)—does not provide a match (Fig. 6). *Achatina fulica* is described as having 7 to 12 whorls with a narrow and conical spire (Bhattacharyya et al. 2014). The Magubike specimens only have four whorls and a stout spire. Furthermore, *A. fulica* should have at least twice the length when compared to the height of the shell (Bhattacharyya et al. 2014), whereas the Magubike shells are closer to a globose shape with similar length and height dimensions.

The shell characteristics of the whole Magubike LSS are more similar to *Burtoa nilotica* (Pfeiffer 1865), than the endemic *A. fulica*; this illustrates the point that relying on the designation of *Achatina* to describe all land snails and beads may be insufficient. *Burtoa nilotica* is described as a savannah species, found across southern and central Africa where they can reach 111–126 mm in length (Crowley and Pain 1959; van Bruggen 1969). The number of whorls is similar to the Magubike specimens, with *B. nilotica* reported having three to five based on photos. The shell dimensions of the Magubike shells, however, are significantly smaller than described for *B. nilotica*, with the average length of full grown Magubike specimen being 86.31 mm (with a height of 61.51 mm). Throughout five seasons of survey and archaeological excavation at the site, we have not observed any living land snails or even evidence of recent snail activity. It is possible that the snail species from antiquity are no longer present in the study region. If, however, snail populations in this area remained relatively consistent over time, these MSA species are a likely candidate for those used in later bead manufacture.

Whatever their taxonomic attribution, the complete shells in the MSA levels are the best proxies available for the production of shell beads found in the rockshelter. Comparing measurements and microstructure of the shells and LSS beads supports this assertion. None of the beads have the distinctive periostracum or prismatic layers present, and none appear to have the shiny inner nacre layer. The absence of these inner and outermost layers suggests that the bead thickness would be noticeably less than that of the original shell wall. The unmodified LSS from Magubike is of sufficient thickness to have produced the recovered beads. Further, the distinctive banding of the cross-lamellar layers present in the beads is absent from the OES and marine shell samples observed.

### Preliminary Conclusions from the Magubike Case Study

The finds from Magubike are a new instance of Iron Age LSS beads, and the first of their kind to be directly dated. Their chronology provides additional perspectives on snail bead development and spread. The range of direct radiocarbon dates and the visually distinct bead styles suggest a long-term use of LSS beads in the region, with potential for stylistic evolution. Although it is not possible at present to identify the raw material to species, clear laminations visible on bead surfaces are consistent with LSS. Furthermore, comparisons with modern and archaeological marine and LSS shell reveal consistent morphological patterns, supporting earlier work by Ward and Maggs (1988). Although a larger sample size is needed to test whether these features are sufficiently unique to form the basis for a formal method, the thickness and microstructure of land snail shells seem to be promising identifiers. Some authors (e.g., Almagro et al. 2016) suggest that detailed chemical analysis of the layers can be used to distinguish between taxa. This line of research is deserving of further investigation. Nevertheless, preliminary work strongly suggests that it is possible to differentiate LSS from OES and other types of shell based on easily observable features of the bead surface and cross section.

## Discussion

A review of published sources, combined with new data from Magubike Rockshelter and other previously unreported sources, presents a startlingly vast picture of LSS bead occurrence in sub-Saharan Africa. Furthermore, this is almost certainly an underestimate given variable reporting standards for land snail artifacts and disc beads respectively, as well as the challenges associated with identifying LSS as a bead raw material. We hope that by highlighting these artifacts, we can promote further discussion and the publication of new data that help refine understanding of the role LSS beads played in prehistory.

The present state of this dataset is nonetheless intriguing. Analyzing the contexts in which LSS beads are found has great potential to contribute to our understanding of ecological, economic, and sociopolitical processes unfolding in the Holocene. There is already much to learn simply from examining when LSS beads occur and their geographical distribution. Ultimately, these patterns are what allow us to begin asking *why* they may have been manufactured at all.

### When Do LSS Beads Occur?

Investigating the chronological range of LSS bead cases is challenging because other than those from Magubike, there are no direct dates on these artifacts. At present, it is only possible to determine which LSS beads have published provenience data and correlate that with associated radiocarbon dates from charcoal and other datable material within the same level or strata. This of course only produces a rough estimate of the antiquity of LSS beads. Expectedly, the range is quite broad, spanning approximately 15,000 calBP to as recently as 160 calBP. However, the upper extent of this range is potentially a significant overestimate.

Only four published sites have LSS beads in strata dated to older than 5,000 BP: Bushman Rockshelter and Tloutle in South Africa, and Kalemba and Makwe Rockshelters in Zambia. At Bushman Rockshelter, half of the *Achatina* beads (*n* = 3 of 6) and all of the broken and unfinished beads (*n* = 3) come from levels 1–5 (Plug 1982, p. 61). Plug (1981, p. 14) notes elsewhere that levels 1–2 show mixing of modern and Iron Age material, including Lydenburg-type potsherds, tobacco leaves, and goat dung. It is therefore plausible that the LSS beads postdate even the more recent date of 11,131–10,717 calBP derived from charcoal in level 2 (Plug 1981, p. 14). Similarly, inverted mid- and late Holocene dates at Tloutle, one of which is from the seventeenth century, imply that at least uppermost levels are “probably a palimpsest of mid-Holocene and recent Holocene occupations” (Mitchell 1993, p. 89). At Kalemba, the only four beads specified to be *Achatina* come from the uppermost horizons R and S, the latter dated to the last 300 years (Phillipson 1973, 1976). The nine remaining beads from earlier P and Q horizons are referred to as “small undeterminable land snail (not *Achatina*),” that are “made from the flat apex of the shell…punched at the thin centre of the spiral” (Phillipson 1976, p. 157). Judging from the line drawing on page 156, it appears that these are examples of pierced opercula rather than shaped disc beads. Finally, it is worth noting that Makwe Rockshelter has an Iron Age occupation with a high density of artifacts, found above bead layers. It is therefore possible that the *Achatina* beads from that site are intrusive, especially given the stratigraphically inconsistent dates reported by Phillipson (1976, p. 72). As beads can be quite vertically mobile, particularly in sites with long occupational sequences and trampled, mixed upper strata, direct dating should be strongly considered before any of these cases can be confirmed as early Holocene.

**Fig. 7:**
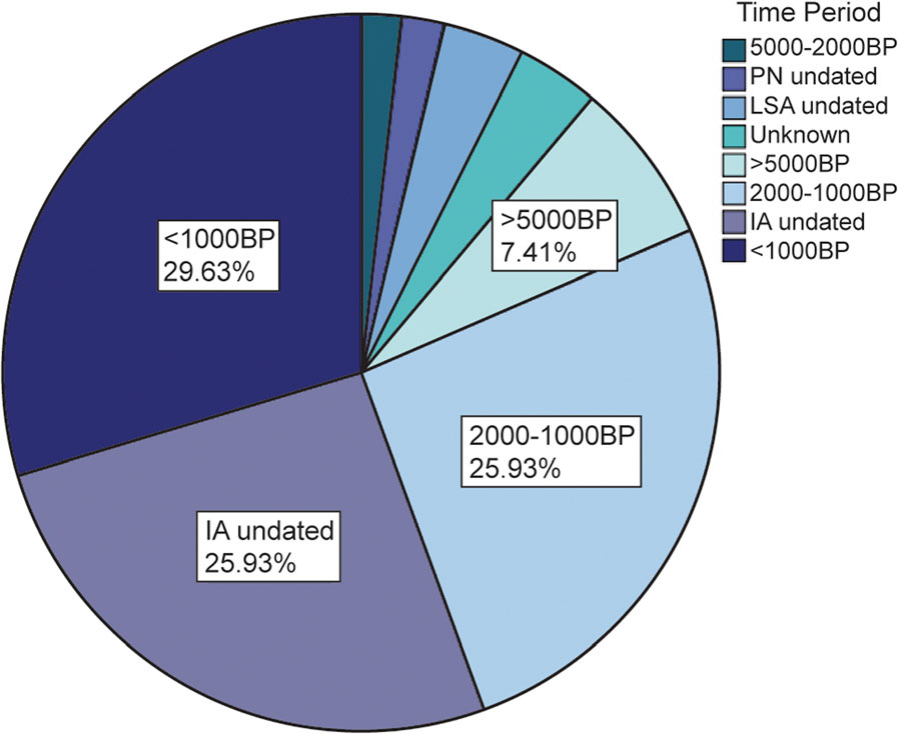
Distribution, by age, of sites with specified *Achatina* or snail shell beads

Otherwise, the vast majority of the remaining cases from Table 1 are associated with Iron Age deposits. When only the sites with specifically *Achatina* or “land snail” beads are considered (*n* = 55 of 81, sites marked as LSS in Table 1), 56% of known LSS beads come from contexts dated within the past 2,000 years. An additional 26% are undated but associated with Iron Age material culture (Fig. 7). This constitutes strong evidence that LSS beads are a relatively recent phenomenon, potentially associated with Iron Age peoples or contact with them.

These preliminary findings concord with anecdotal observations found throughout the archaeological literature on LSS beads. Ward and Maggs (1988, p. 407) claim that while OES was preferred for beadmaking in the LSA, Achatinidae dominated the early Iron Age. Garlake (1976, p. 41) adds that shells beads of both OES and *Achatina* material “occur in all Later Iron Age sites in eastern Africa,” indicating continued use throughout this period. Both Mazel (1999), and Hall and Smith (2000) suggest that LSS beads are associated with agriculturalists and may not have been prioritized before the spread of farming. Yet, when reporting LSS beads at the potentially nineteenth-century site of Ficus Cave, Partridge (1966, p. 131) singles them out as the only find that would not be expected in most modern Bantu villages. Formally testing the association between LSS beads and the Iron Age, and determining when this tradition appeared and (potentially) disappeared, requires further research into the archaeological context of individual cases as well as direct radiocarbon dating of the artifacts in question. Based on this initial review, however, it appears that LSS beads appear over 40,000 years after OES traditions are established across sub-Saharan Africa and may persist until the recent past if not ethnographic present.

### Where Do LSS Beads Occur?

Consideration of where LSS beads occur provides additional insight into factors that may have shaped their creation. The earliest examples, and the greatest number of individual sites, are found in the southernmost part of the continent. Forty percent of all sites with specified LSS beads are found in South Africa (*n* = 22 of 55 LSS sites, Table 1) followed by Zimbabwe (*n* = 8), Botswana (*n* = 7), and Zambia (*n* = 6). Tanzania is also well represented (*n* = 7), although half the confirmed cases come from this study, suggesting further work is needed in the region. Curiously, there is no compelling evidence of LSS beads in western Africa. We found only one potential case from Kintampo Rockshelter No. 6 in Ghana; however, Stahl (1985, p. 138) suspects the “small shell beads” are more likely from marine Cerithids because the land snail and fresh water mollusc shells in the deposits were too thin to have produced the observed beads.

Concentration of LSS beads in Southern Africa can be at least partly attributed to greater archaeological research focus. Recent work on the Middle and Later Iron Age in Tanzania has revealed LSS beads at dozens of lesser known sites (J. Walz, pers. comm.), suggesting gaps remain in our knowledge of their distribution. However, we cannot dismiss the fact that the four-corner region of southern Africa was the location of successive large-scale complex societies during the Iron Age, with populous capitals such as Mapungubwe, K2, and Great Zimbabwe (Huffman 2009; Monroe 2013). Since such polities were heavily integrated in coastal-interior trading networks, and beads are an important social signaling mechanism in societies undergoing processes of vertical stratification, it is understandable that LSS beads might appear more frequently. Southernmost Africa is also known for ethnographically documented networks of *hxaro* exchange among !Kung-speaking hunter-gatherers in which OES beads play a major role (Mitchell 1996; Wiessner 1982). Social, political, and economic developments in the later Holocene of this region probably contributed to increased bead demand among both food producers and foragers, with farther ranging effects along trade routes. The presence of Zhizo series glass beads in early strata at Kwa Mgogo suggests that these networks had reached as far as northeastern Tanzania by the mid-tenth century AD, perhaps also explaining the presence of LSS beads in the region (Walz 2010a; Walz and Dussubieux 2017).

Plotting the coordinates of known sites (refer again to Fig. 1) reveals another pattern: LSS bead sites cluster in the Afromontane belt along the eastern extent of the continent even though land snail species occupy a much broader range. Land snails are found from sea level to over 3,000 m in elevation and can live in a wide variety of biomes from coastal shores to savannah grassland to rainforest and mountainous range (Boxnick et al. 2015; Chukwuka et al. 2014; Rowson et al. 2011). If LSS raw material was more widely available, why were LSS beads seemingly restricted geographically?

The eastern distribution of documented LSS bead use does not appear to be related to environmental factors proposed to affect shell growth and form, such as moisture, temperature, substrate composition, predation, or population density. Although thinner shells can result from environmental calcium deprivation, the growth of snail shells is not well understood (Oosterhoff 1977; Owen 1965). There is some correlation between moist environments and shell morphology, with wetter (and warmer) environments contributing to an elevated growth rate through increased activity and feeding, producing larger shells to accommodate large snail bodies. However, both large and small snail species can occur in the same environment so an environmental explanation for shell bead production seems unlikely (Goodfriend 1986). Human intervention is another possibility; agricultural activity around the Limpopo-Shashi confluence in the last 2,000 years may have encouraged snail consumption of crops and thus snail collection to curb these invasive agricultural pests (see Mead 1961).

The lack of LSS bead sites reported in central and western Africa remains particularly confusing. This area is presently inhabited by *A. achatina*, one of the three largest LSS on earth (Awodiran et al. 2015). Ethnographic literature from these regions points to *A. achatina* as a source of food (Fagbuaro et al. 2006; Mead 1961; Walz 2017), suggesting the snails were a familiar resource to people from those areas. Furthermore, given the impressive size of *A. achatina*—150–200 mm in length and up to 100 mm in height (Bequaert 1950)—it should be a good candidate for beadmaking raw material. It is possible the absence of published data may reflect biases in collection and reporting and not necessarily a dearth of LSS beads. However, the distribution of such large snail species in the past requires further study. Evaluating what LSS species make good bead raw materials and their ancient distributions would help determine functional and geographical constraints on societies making beads.

### Why Do LSS Beads Occur?

Drawing on the evidence of when and where LSS beads are known thus far, we can engage with hypotheses to explain *why* they were created. It is perhaps easier to start by eliminating several possibilities. Ancient peoples did not start making LSS beads because they lost the ability or preference for making beads out of OES, which continue to be prevalent into the ethnographic present. Conversely, LSS beads are not absent earlier in the record because of a dearth of land snails. The sheer amount of unmodified snail shells in archaeological deposits across the continent, combined with evidence for snail middens at sites like Mumba (Mehlman 1979), implies prehistoric peoples were familiar with the potential utility of snail shell. Although there may be taphonomic explanations for why LSS beads are not typically found in pre-Iron Age contexts, preservation should not be a major factor. Land snail shell is less brittle than OES and has been described as more durable (Bvocho 2005; Hanisch 1980). Bias is more likely to be introduced by the underreporting of these finds.

Alternative explanations are rooted in the environmental, economic, and sociopolitical context of this time period in eastern and southern Africa. Although few sources discuss why LSS beads are present beyond the immediate questions of site formation, hypotheses for their creation tend to revolve around several interrelated themes: environmental change, population increase and incipient social stratification, and the elaboration of Iron Age trade networks.

#### Environmental Change

The appearance of LSS beads may be linked to environmental change causing the displacement and scarcity of ostriches (Hall and Smith 2000; Mitchell 1993). There has been considerable discussion on the connection between climate change and the spread and development of Iron Age cultures around the Shashi-Limpopo Basin (Holmgren and Öberg 2006; Huffman 1996, 2008; Tyson et al. 2002; Tyson and Lindesay 1992) and along the Swahili coast (Walz 2010a). Warm wet periods around c. 2,000 BP and c. 1,000 BP are implicated in the spread of Iron Age populations from drier eastern Africa and population increase associated with K2 and Mapungubwe, while the Little Ice Age c. AD 1,300 may have influenced the abandonment of Mapungubwe and the rise of Great Zimbabwe (Huffman 1996, 2008). Environmental change and associated human responses could have impacted ostrich ranges. Hall and Smith (2000, p. 36) argue that the Zhizo occupation of the Limpopo belt during a period of increased rainfall displaced ostriches into more marginal areas to the west. Mitchell (1993, p. 123) makes a similar argument for the reduction of personal ornaments at Tloutle after 6,140 BP, suggesting that ostrich populations may have declined in the basin toward the mid-Holocene. During favorable climatic periods, positive feedback between agricultural land cultivation and rising population densities would have also pushed ostriches and other wild fauna to the margins of large settlements. Increasing reliance on domestic species diminishes natural animal and plant diversity and tends to push wildlife away from habitation sites (Plug 2000, p. 122).

Other explanations for LSS bead production focus on raw material availability on the local scale. These arguments tend to center on whether ostrich and land snail populations can survive in the same environments or if prevalence of one type of bead over the other can be used as a proxy for environmental conditions. Maggs and Ward (1984, p. 124) argue that ostriches and snails are “ecologically incompatible,” with the former requiring savanna environments and snails requiring forest and brush because they are “quickly killed by direct sunlight.” This is not strictly true since land snails are nocturnal and can survive in the same environments as ostriches by hiding and/or resting during the day, perhaps under brush or in rockshelters, and coming out at night when conditions are more favorable. Both types of organisms would be drawn to ephemeral fresh water resources, although ostriches can obtain the majority of their water requirements from eating plants (Bertram 2014). It is not quite so straightforward to use the presence of one type of shell to infer the rarity of the other.

#### Population Increase and Incipient Social Stratification

Related to discussion of environmental fluctuations and ostrich scarcity, production of LSS beads may have been a response to increased demand for beads. Heightened population densities are detectable beginning in the early Holocene, with associated elaboration of material culture including increased production of beads (Cox et al. 2009; Dayet et al. 2017; Mitchell 1996). This process continued throughout the Holocene, aided by the spread of food production throughout many parts of the continent. The development of Iron Age polities in southern Africa is associated with localized population increases around the Shashi-Limpopo Basin, coupled with increased agricultural production. Population concentration and rising social complexity led to the formation of elites and further increases in craft production. Caches of OES and LSS beads and bead blanks at Schroda are considered to be evidence for elite control of craft production (Hall and Smith 2000; Hanisch 1980). Bead wearing is an important form of social signaling in many African societies and beyond, relaying information about the wearer’s wealth, control over labor, and/or status through non-verbal means (Bvocho 2005; Klehm 2017). Increased population density coupled with emerging vertical stratification likely increased economic demand for beads and therefore bead raw materials. If OES became more difficult to procure, either through localized environmental shifts or elite-controlled access, LSS may have produced a viable alternative to keep up with markets.

However, there is some tension in the literature regarding the relationship between OES and LSS, and if the latter represent higher status, exotic products or lower status, locally made alternatives. Arguments for LSS beads as prestige goods are based on the rarity of snails in the immediate vicinity combined with a lack of on-site manufacture debris, indicating beads were traded in in their finished form. Klehm (2013) and Klehm and Ernenwein (2016) interpret LSS beads as higher value trade items at the Middle and Late Iron Age sites of Khubu la Dintša and Mmadipudi Hill, Botswana, relative to locally produced OES beads. Dubroc (2010) draws similar conclusions for the nearby center of Bosutswe, where LSS beads are concentrated in levels with other exotic goods such as mussel shell beads and non-local game. While the value and status of these artifacts would have been historically contingent, LSS like other non-local beads were seemingly used to establish, maintain, and communicate relationships and social influence within emerging state societies (Walz 2010a; Klehm 2017).

Calabrese (2000), however, argues for the lower status of LSS relative to OES beads at Leokwe Hill, South Africa, where elite Leopard’s Kopje individuals occupied a hilltop above Zhizo commoners. The elite hilltop, area A, revealed higher densities of glass beads (33% of total number) and OES beads (62%) than the commoner area B. Notably, only 1% of *Achatina* beads found at the site come from area A, whereas 73% come from area B (Calabrese 2000, p. 202). Calabrese (2000) interprets these patterns as reflecting differential access to various types of material culture, and potentially raw materials, with commoners having decreased access to glass and OES beads. Given the visual similarities of OES and LSS beads, particularly from a distance, it is tempting to think of the latter’s appearance as reflecting prehistoric counterfeit operations. No blanks or preforms are reported at Leokwe Hill, so such activities must have taken place elsewhere.

On-site manufacture of LSS beads, in the form of unfinished preform stages, are only reported at a small number of sites: Mutshilachokwe in Zimbabwe (Manyanga 2006), Makwe in Zambia (Phillipson 1976), Gede in Kenya (Flexner et al. 2008), Kwa Mgogo, Gonja Kalimani and Gonja Maore in Tanzania (Walz 2010a, 2017), Taukome in Botswana (Denbow 1983), and Magogo and Bushman Rockshelter in South Africa (Hanisch 1980; Maggs and Ward 1984; Plug 1982). Overall status of these items was likely fluid depending on place of manufacture, availability of land snail shell for raw material, and finesse of the final product. Although the existence of LSS beads is almost certainly tied to expressing some form of status or identity, this is contingent on context and not easily generalized in the literature.

#### Iron Age Trade Networks

Questions of exotic vs. local items play into other discussions about the role of LSS in trade. Growth of complexity in southern Africa is partly attributed to the development of elaborate trade networks between the Indian Ocean and the interior (Calabrese 2000; Huffman 1972). Establishment of trade routes and a market for the circulation of personal ornaments, including copper bangles, gold, and glass trade beads, is inextricably linked to other processes discussed such as agricultural intensification, population increase and the emergence of nascent elites, and perhaps ecological changes affecting availability of other raw materials such as OES. LSS beads were potentially incorporated into these networks, meeting existing demand for shell beads founded on long-standing traditions using OES. It remains unclear whether Magubike Rockshelter, located more than 1800 km from major centers in the Shashi-Limpopo Basin, would have been part of the same networks or involved in more localized trade. The presence of LSS and Zhizo series glass beads at Kwa Mgogo only 450 km away suggests both scenarios are possible (Walz and Dussubieux 2017). It is noteworthy that the two clusters of LSS beads at Magubike date to nearly 1,000 years apart (~ 500 years before and after the Kwa Mgogo finds), suggesting considerable time depth for these artifacts in eastern Africa, and potentially independent innovation. Further data on the prevalence of LSS beads in eastern Africa, as well as evidence for movement between eastern and southern Africa, are needed to better assess trade interconnectivity between these regions.

It is also interesting to consider the role interaction between hunter-gatherer and agropastoralist populations played in the appearance and proliferation of these artifacts (see Wilmsen and Denbow 1990 and comments for consideration of political economies in the Kalahari, past and present). OES beads are one of the primary components of *hxaro* exchange networks among Khoesan groups such as the Zu/’hoãsi. Individuals are linked in ongoing reciprocal exchange relationships with *hxaro* partners, circulating various types of gifts (except food), although OES beadwork is preferred (Mitchell 1996; Wiessner 1982). Ethnographic and archaeological data demonstrate the Khoesan also traded ivory, skins, ostrich feathers, and copper with other ethnic groups (summary in Mitchell 1996). Hall and Smith (2000) hypothesize that foragers engaged in surplus bead production for trade with farmers, although this may have been limited to raw materials with manufacture located and controlled by the elite within cities. Mitchell (1996, p. 71) considers the possibility that foragers were engaged in simultaneous bead production for multiple purposes. This was previously documented by Wiessner, who observed specialized OES manufacture of smaller beads for *hxaro* and larger beads for trade (cited in Mitchell 1996, p. 71). In this scenario, perhaps it was the LSS beads that were being manufactured (or raw material supplied) to agropastoralists to maintain forager supplies of OES for *hxaro*.

## Conclusions

At present, it appears that LSS beads are largely confined to eastern and southeastern Africa during the last 2,000 years, associated with Iron Age communities. Although this is partly biased by ongoing research foci within southeastern Africa, there is compelling evidence that this phenomenon is linked to population increase, elaboration of trade networks, and the growth of social complexity during the later Holocene. It is intriguing that LSS beads appear tens of thousands of years after the establishment of OES beadmaking, despite the much longer tenure of land snails in the same parts of the world. Although we offer some possible explanations as to why this might be, more data are required to begin formally testing hypotheses. Highlighting these artifacts is intended to promote discussion, and ultimately publication, of additional finds from scholars working in diverse regions across the continent. We hope that re-examination of many disc bead assemblages reveals some unexpected finds.

This exercise in reviewing cases of LSS beads in sub-Saharan Africa has perhaps revealed more about what we do not know than what we do know. Once geographic and temporal distributions are better defined, there are many fruitful directions for further research. For example, LSS bead production remains largely a mystery. Although there are numerous ethnographic accounts of OES beadmaking, it is more difficult to find accounts using LSS. Several site reports note the presence of LSS preforms, but the lack of high-quality images, drawings, or descriptions render it impossible to assess production stages between sites or through time. Since both OES and LSS are naturally occurring convex shells of comparable thickness, both would be broken into smaller pieces, drilled and shaped, and likely followed similar production sequences (see Kandel and Conard 2005; Orton 2008). Walz (2010a, p. 211) notes that unfinished preforms were often drilled from the inner surface of the shell, which is consistent with OES techniques suggesting a procedural similarity. However, variability between LSS and OES manufacture techniques (particularly the application of drilling and shaping with different shell characteristics), and variability among LSS examples, remains unexplored.

Greater attention to distinguishing LSS beads from OES, direct dating of known and new cases, and reportage using standardized terminology should help increase visibility of these finds and pave the way for future work. To this end, we have offered a review of cases known thus far and practical approaches to identifying LSS using the easily observable microstructure of shell profiles. Recently excavated LSS beads with direct dates from Magubike Rockshelter illustrate these methods and add an important data point to the distribution of LSS beads across eastern and southern Africa. We hope this preliminary work prompts additional research questions on related topics, such as differing manufacture techniques between LSS and OES beads, the co-occurrence of these artifacts with other types of personal ornamentation and symbolic imagery, and the broader role of LSS beads in prehistoric African societies.
